# PixelDeck: A local-first media library manager for biomedical imaging

**DOI:** 10.1016/j.softx.2026.102803

**Published:** 2026-06-13

**Authors:** Benjamin L. Kidder

**Affiliations:** a Department of Oncology, Wayne State University School of Medicine, Detroit, MI, USA; b Karmanos Cancer Institute, Wayne State University School of Medicine, Detroit, MI, USA

**Keywords:** Histology imaging, Fluorescence microscopy, High-throughput imaging, Image management, Digital pathology, Scientific software

## Abstract

Biomedical imaging workflows generate large collections of histology, fluorescence microscopy, and high-throughput imaging assets that are difficult to organize, search, and reuse using conventional file systems. We present PixelDeck, an open-source, local-first browser application for management of large image and video libraries on commodity workstations. PixelDeck integrates recursive import, SHA-256-based duplicate detection, metadata extraction, thumbnail generation, and full-text search within a responsive interface. The system uses a transparent SQLite-backed architecture with managed filesystem storage and asynchronous processing. Benchmarks on fresh isolated libraries containing up to 1000 assets showed import throughput exceeding 500 files/min and representative median query latencies in the millisecond range.

## Metadata

C1: v0.5.0

C2: https://github.com/KidderLab/PixelDeck

C3: 10.5281/zenodo.19612239

C4: MIT License

C5: Git

C6: TypeScript, Next.js, React, Prisma, SQLite, Sharp, FFmpeg

C7: Node.js ≥20, pnpm ≥9, SQLite, FFmpeg/ffprobe

C8: https://github.com/KidderLab/PixelDeck

C9: benjamin.kidder@wayne.edu

## 1. Introduction

### 1.1. Motivation and significance

Biomedical imaging has evolved into a data-intensive discipline, with workflows in histology, fluorescence microscopy, live-cell imaging, and high-throughput screening routinely generating tens to hundreds of thousands of derived image and video assets per project [1–5]. These assets typically include processed outputs such as segmentation overlays, multichannel composites, time-series snapshots, quality-control images, and model-generated predictions. While primary acquisition systems and analysis pipelines are often well defined, the downstream management of these derived datasets remains largely ad hoc.

In practice, imaging outputs are stored across nested directory structures on local disks, network storage, or external drives, often with inconsistent naming conventions and minimal metadata standardization [1–3,6–8]). This leads to several persistent challenges: (i) inefficient retrieval of previously generated results, (ii) inability to identify and eliminate duplicate assets, (iii) difficulty in assembling reproducible subsets for downstream analysis or figure preparation, and (iv) limited reuse of historical datasets for comparative or machine learning applications. As datasets scale, these issues become a major bottleneck in day-to-day research workflows [1–3,6–9].

Existing solutions do not fully address this problem space [6–13]. Lightweight approaches based on filesystem browsing lack indexing, search, and deduplication capabilities. At the other extreme, enterprise-scale platforms and laboratory information management systems provide structured data models but require substantial infrastructure, configuration, and domain-specific integration [6–10]. More recent digital pathology and microscopy tools, including SlideRunner, QuPath, and Fiji/ImageJ plugin ecosystems, provide important capabilities for whole-slide annotation, pathology analysis, or batch image processing [11–13]. However, these tools are not primarily designed as local media libraries for deduplicating, indexing, browsing, and exporting heterogeneous collections of already generated image and video assets.

PixelDeck was developed to address this unmet need by providing a local-first, open-source platform for managing large collections of derived imaging assets [14]. The design focuses on reproducible, metadata-aware organization and rapid visual exploration of image libraries on commodity workstations, without requiring cloud infrastructure or centralized services. By integrating content-based deduplication, structured indexing, and responsive browser-based interaction, PixelDeck provides a workstation-oriented alternative to heavier server- or cloud-based image-management platforms for exported biomedical media.

PixelDeck uses a modular architecture consisting of a Next.js frontend, a SQLite-backed persistence layer via Prisma, and a background worker for asynchronous processing. The system separates immutable original files from derived assets such as thumbnails and previews, ensuring reproducibility and efficient browsing.

### 1.2. Software description

PixelDeck is a local-first software application for organizing, browsing, and exporting large image and video libraries on commodity workstations [14]. It is designed for biomedical imaging workflows in which histology images, fluorescence microscopy outputs, and high-throughput imaging derivatives have already been exported into standard viewable formats and must be managed efficiently for downstream review, curation, and reuse. The software combines a browser-based user interface, a lightweight API and metadata backend, managed local storage, and a background worker for long-running processing tasks. This design allows PixelDeck to remain responsive during large imports and exports while preserving transparent, reproducible local control of both metadata and media files.

#### 1.2.1. Software architecture

PixelDeck follows a modular, local-first architecture composed of four principal components: a browser-based presentation layer, an application/API layer, a persistence layer, and an asynchronous worker layer. The presentation layer is implemented with Next.js and React and provides the main interface for browsing, searching, filtering, selecting, and exporting assets. To support large libraries, the interface uses virtualized grid and table views, progressive loading, and lightweight derived media rather than full-resolution originals in the main viewport.

The application layer is served through Next.js and exposes REST-style HTTP API routes for assets, imports, browser uploads, exports, media retrieval, folders, collections, settings, tags, and health checks. These routes are used by the browser interface and can also serve as integration points for local automation. For command-line ingestion, the repository provides the folder-import utility shown in Section 2.3, which creates an ImportJob record that is processed asynchronously by the worker. PixelDeck does not currently expose a gRPC interface.

The persistence layer combines SQLite for structured metadata with a transparent filesystem layout for original and derived media. Prisma is used as the object-relational access layer between the application code and SQLite. The schema is centered on the Asset model and is extended by tables for tags, collections, import jobs, duplicate events, export jobs, saved searches, and application settings. PixelDeck also maintains a SQLite full-text search index over filenames, display names, sources, tags, and aggregated searchable text to support efficient retrieval.

**Fig. 1.**
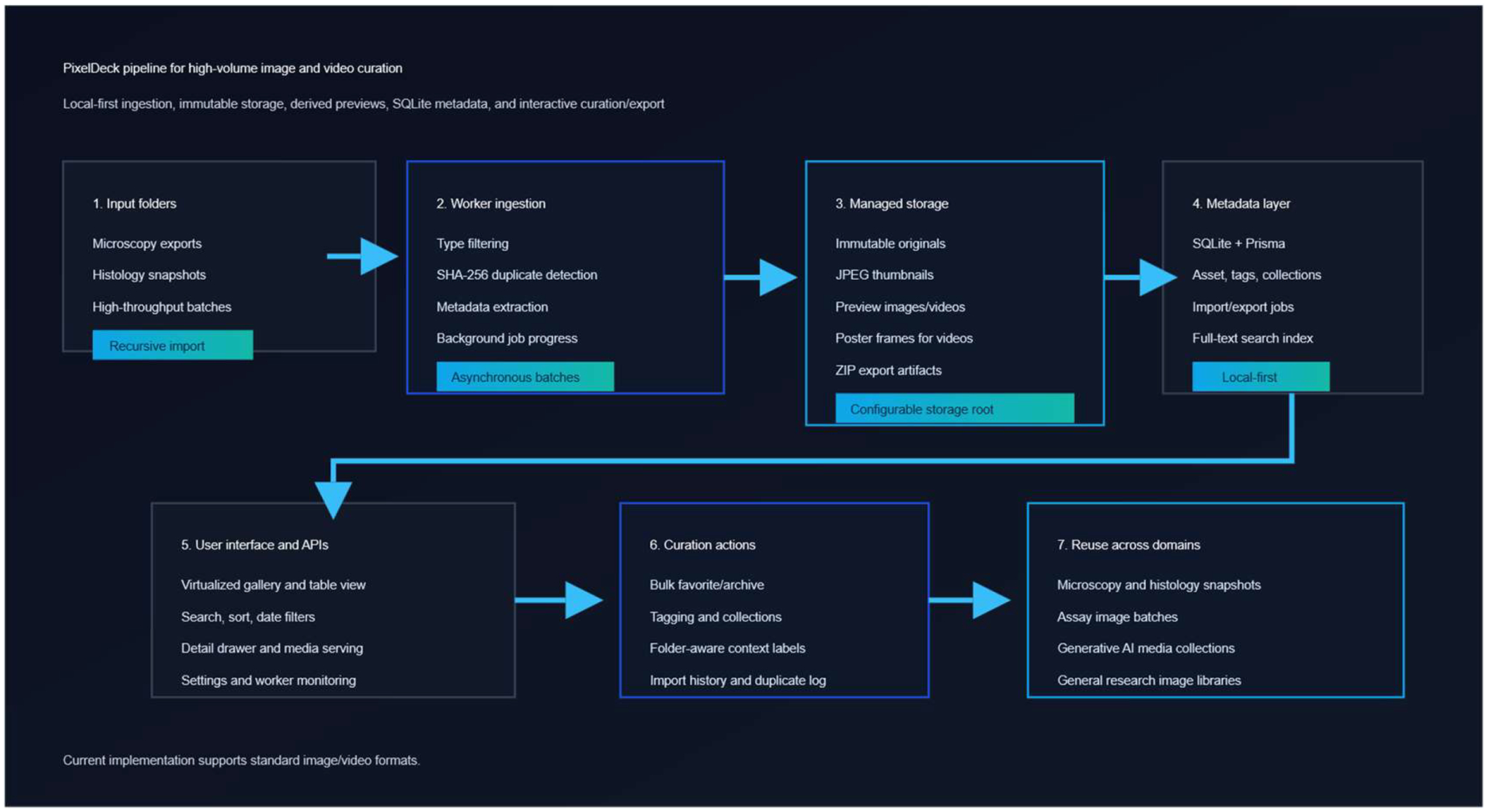
PixelDeck software architecture and processing pipeline. External imaging folders are imported into managed local storage, processed by a background worker for duplicate detection, metadata extraction, and derivative generation, indexed in SQLite with full-text search, and exposed through the browser-based interface for browsing, curation, and export.

Managed media storage is organized into dedicated directories for immutable originals, thumbnails, previews, video poster frames, ZIP exports, and logs. This separation preserves data integrity while allowing efficient rendering and straightforward migration or backup. Computationally expensive operations are delegated to a dedicated worker process, which polls for pending import and export jobs, processes media in batches, updates job state in the database, and keeps the interactive application responsive during long-running operations. A schematic overview of the PixelDeck software architecture and processing workflow is shown in Fig. 1.

#### 1.2.2. Data model and managed storage

PixelDeck provides several major functionalities for large-scale image library management. First, it supports recursive import of image and video files from local folders or external drives through either a browser upload workflow or a command-line import utility. During import, the software performs duplicate detection using SHA-256 hashing, copies non-duplicate originals into managed storage, extracts metadata, and generates thumbnails, previews, and video poster frames for efficient display.

Second, PixelDeck provides interactive browsing and retrieval through virtualized grid and table views, free-text search, metadata-aware filtering, sorting, and detailed per-asset inspection. These functions are intended to support rapid review of large collections without requiring manual traversal of deeply nested filesystem directories.

Third, the software supports curation-oriented actions including selection of individual or multiple assets, assignment to collections, marking of favorites, archiving, and monitoring of import history. Fourth, PixelDeck supports asynchronous ZIP export of selected assets, allowing users to prepare subsets of images or videos for downstream analysis, collaboration, or figure preparation without interrupting ongoing browsing.

Together, these functionalities allow PixelDeck to act as a curation and retrieval layer for histology, fluorescence microscopy, and high-throughput imaging workflows based on exported media files.

#### 1.2.3. Sample code snippets analysis

A representative PixelDeck command-line workflow begins by creating a recursive import job: pnpm import:folder – –folder “D:\Imaging\Histology\2026” –source “histology-2026”

This command does not perform the full import synchronously. Instead, it records an import job in the local database, allowing the software to process the dataset asynchronously. This design is important for large biomedical image libraries because it decouples user interaction from long-running file operations.

Background processing is then handled by the worker: pnpm worker

The worker continuously polls for pending import and export jobs, computes file hashes for duplicate detection, extracts media metadata, generates derived assets, updates the search index, and records job progress. This explicit separation between job creation and job execution is central to PixelDeck’s architecture and allows the browser interface to remain responsive during sustained processing of large folders.

For performance benchmarking, we generated fresh isolated PixelDeck libraries containing 100, 500, and 1000 image assets sampled from the existing PixelDeck image corpus and imported each subset into a clean temporary database and storage root. Import duration, throughput, and peak memory usage are reported in Table 1, and representative query latencies are reported in Table 2. Benchmark input characteristics, including file size, image dimensions, and dominant file formats, are summarized in Table S1. Across the three benchmark subsets, mean file size ranged from 0.55 to 0.66 MB, mean image width ranged from 1338 to 1833 pixels, mean image height ranged from 1027 to 1378 pixels, and the dominant formats were JPEG and PNG.

To contextualize PixelDeck relative to representative existing platforms, we added a structured comparison covering deployment model, server requirements, repository behavior, indexing/search support, duplicate handling, and intended workflow scope (Table 3), with additional setup and deployment details provided in Table S2. Fig. S1 further summarizes the qualitative positioning of PixelDeck relative to these representative tools. Because PixelDeck, OMERO, QuPath, Fiji/ImageJ, and BisQue target overlapping but non-identical use cases, we treated this comparison as architectural and workflow-oriented rather than as a one-to-one speed contest.

To strengthen reproducibility, we added a documented Windows quick-start workflow and a bundled example dataset path that can be executed directly from the repository root. We generated a small synthetic RGB sample dataset (n = 12) and verified an end-to-end workflow consisting of dataset generation, folder import under a unique source label, source-filtered browsing, source-filtered search, and duplicate-aware re-import. Verifiable workflow outcomes are summarized in Table S3, and workflow timings are summarized in Fig. S2.

To complement the system-design description with a practical user-oriented example, we added a workflow case study based on the reproducibility sample library. This case study followed four common local curation tasks: recovering an imported subset, searching within that subset, re-importing the same folder to verify exact-duplicate handling, and exporting the curated subset as a ZIP archive. For each task, we recorded measured PixelDeck execution time and compared the number of PixelDeck actions with an illustrative manual filesystem workflow performing the equivalent task. The resulting step-comparison and timing summaries are reported in Table S4 and Table S5 and visualized in Fig. S3.

#### 1.2.4. Illustrative example

A representative use case for PixelDeck is the management of exported image collections generated during histology, fluorescence microscopy, and high-throughput imaging experiments [1–3,5]. In these workflows, investigators often produce large numbers of derived image files, composite panels, segmentation overlays, quality-control snapshots, and short videos that must be reviewed and organized after primary acquisition and analysis. These files are commonly distributed across nested folders on local drives or external storage devices, making later retrieval and comparison inefficient.

In a typical PixelDeck workflow, a user imports a parent directory containing one or more experimental subfolders. Each import is registered as an ImportJob and processed asynchronously by the worker. The worker recursively scans the source directory, identifies supported media files, computes SHA-256 hashes for duplicate detection, and copies non-duplicate originals into managed immutable storage. Metadata are then extracted from each file, and derived representations such as thumbnails, previews, and video poster frames are generated for efficient interactive browsing. Asset records and full-text search entries are inserted into the local SQLite database, allowing imported media to become immediately searchable and filterable.

To make this sequence more tangible for readers, we added a step-by-step workflow diagram emphasizing that PixelDeck ingestion is source-agnostic for supported local image and video folders. The diagram follows folder import, exact-duplicate detection, metadata extraction, browsing and filtering, selection/export, and downstream curation actions such as tagging or collection assignment, without requiring any dataset-specific input structure (Fig. S4).

**Fig. 2.**
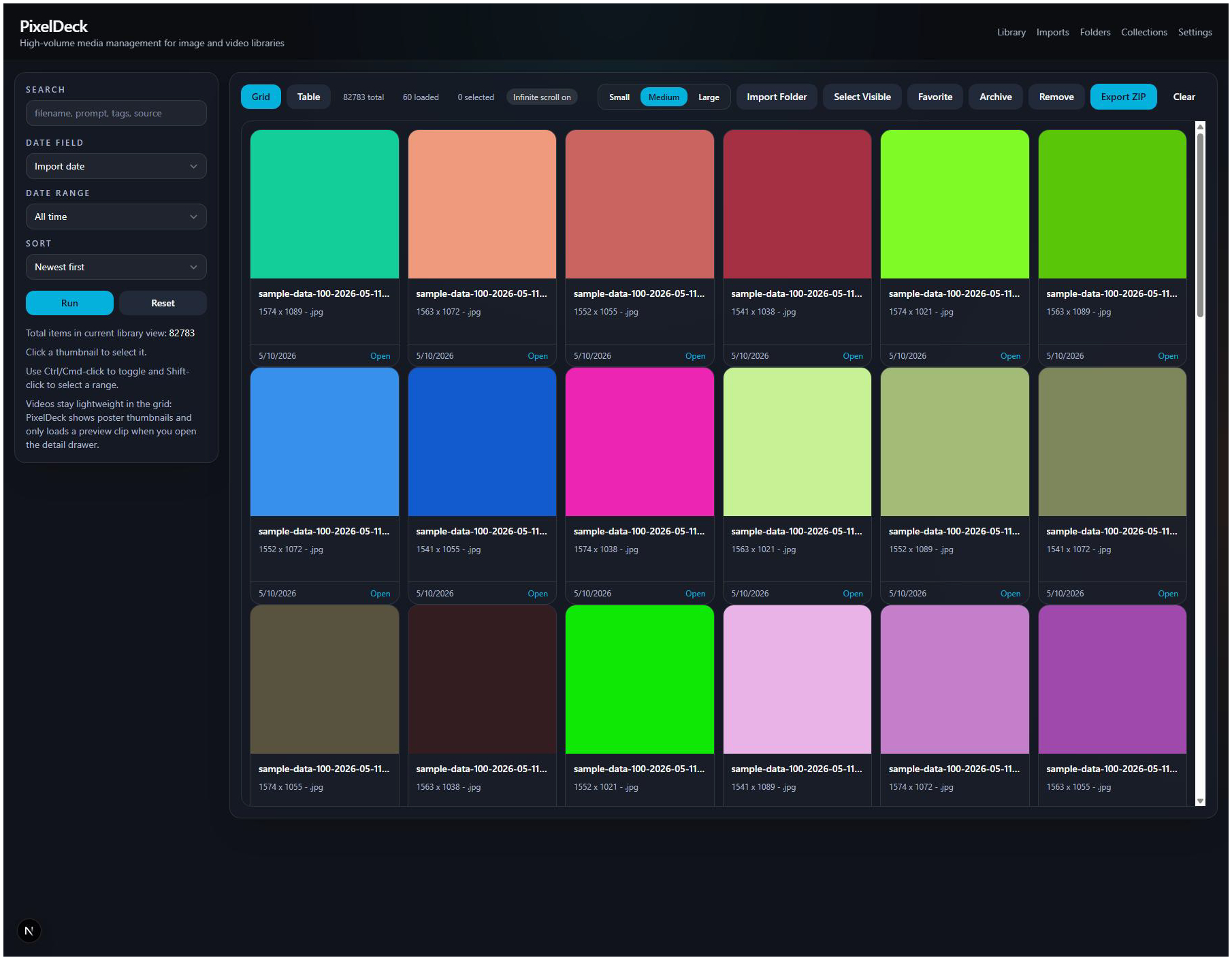
PixelDeck browsing interface for biomedical imaging collections. The populated library view shows the left-side search and filtering panel, virtualized thumbnail grid, item counts and loaded-library status, density controls, selection and bulk-action controls, and ZIP export action used to review and export imported assets. The interface supports metadata-aware filtering, progressive loading, selection, and export of imported image and video collections.

Once indexed, the dataset can be explored through PixelDeck’s main interface using either a virtualized grid view or a tabular view, as illustrated in Fig. 2. Investigators can search by filename, source, folder context, or other searchable fields, sort by import or capture date, and inspect individual items in the detail drawer. This is particularly useful in imaging projects where similar visual outputs may be regenerated across multiple processing rounds or where results from several experiments must be compared side by side. Duplicate-aware import prevents accumulation of redundant files, while collections and ZIP export allow users to assemble subsets for downstream analysis, figure preparation, or transfer to collaborators.

For example, a laboratory may maintain folders of fluorescence microscopy exports containing subfolders for different experiments, imaging runs, or analysis batches. Rather than manually browsing these directories in the operating system, the entire yearly folder can be imported recursively into PixelDeck. The resulting local library allows rapid visual review across experiments while preserving transparent file-level provenance and access to the underlying originals. In this setting, PixelDeck serves as a curation and retrieval layer that reduces friction between image generation and downstream interpretation.

**Fig. 3.**
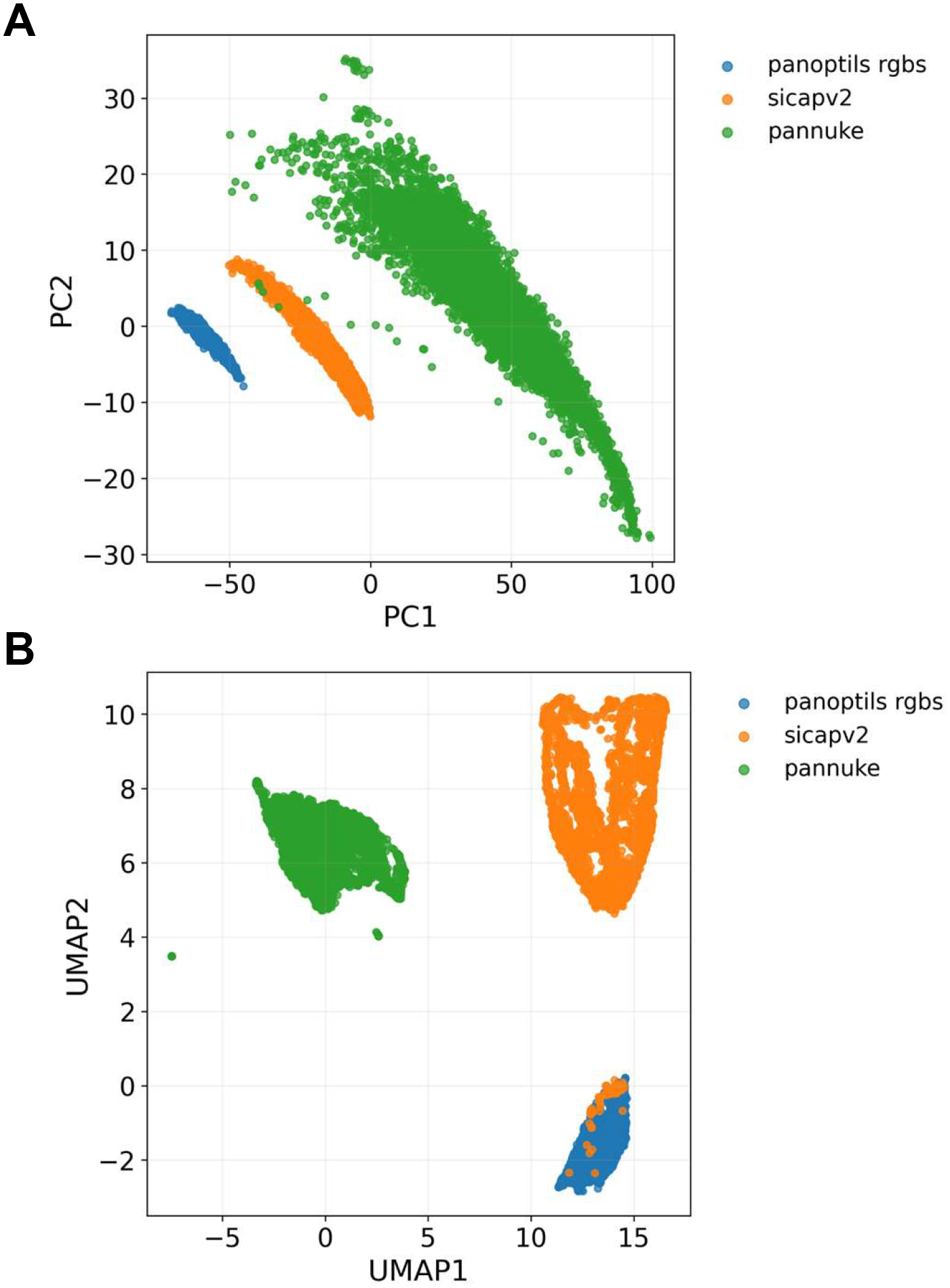
Post-ingestion dataset-profiling example using filtered RGB histopathology collections curated in PixelDeck. (A) PCA of 2353-dimensional RGB descriptor vectors generated from imported PanopTILs RGB, SICAPv2, and PanNuke image sets after source-aware filtering and export. (B) UMAP projection of the same filtered descriptor matrix using cosine distance (n_neighbors=10, min_dist=0.01). The descriptors and projections were generated by an external analysis script after PixelDeck ingestion/export and are not native PixelDeck browser or worker outputs.

To illustrate one optional downstream use case enabled by PixelDeck after local ingestion and source-aware curation, we compared imported public histopathology collections in a quantitative feature space using PanopTILs RGB tiles together with SICAPv2 and PanNuke image datasets [15–17]. This analysis is not intended as a benchmark of browser performance, indexing behavior, or other core software characteristics; rather, it demonstrates how image collections curated in PixelDeck can be organized by source and exported for exploratory dataset profiling after ingestion. The 2353-dimensional image descriptors and PCA/U-MAP projections were generated by an external analysis script applied to PixelDeck-organized exports, not by the PixelDeck browser interface or ingestion worker. The analysis included 14 211 images in total (3026 PanopTILs RGB, 5000 SICAPv2, and 6185 PanNuke) and 2353 combined image-feature dimensions. Under this restricted comparison, PCA showed strong dataset-level structure with PC1 explaining 75.6% of total variance, and UMAP likewise resolved three visually distinct clusters. These results indicate that the earlier PanopTILs overlap was driven primarily by internal heterogeneity within the PanopTILs collection rather than by an inability of the descriptor set to distinguish PanopTILs RGB tiles from SICAPv2 and PanNuke image sets, as shown in Fig. 3.

**Fig. 4.**
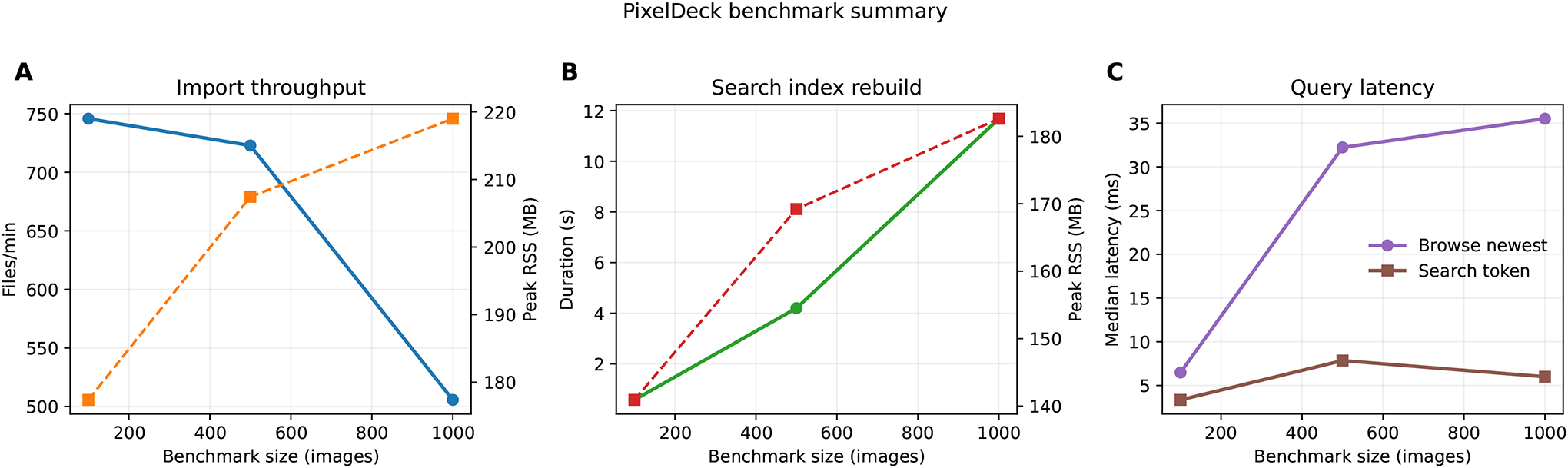
PixelDeck performance benchmarks across fresh isolated image-library sizes. (A) Import throughput and peak resident memory during recursive import of 100, 500, and 1000 image assets into clean temporary PixelDeck libraries. The blue solid line reports import throughput on the left y-axis, and the orange dashed line reports peak resident memory on the right y-axis. (B) Search-index rebuild duration and memory usage across the same benchmark sizes. The green solid line reports rebuild duration on the left y-axis, and the red dashed line reports peak resident memory on the right y-axis. (C) Representative median query latencies for browse-newest and token-search tasks. Across the fresh benchmark series, import throughput exceeded 500 files/min, search-index rebuild throughput exceeded 5000 assets/min, and representative median query latencies remained below 40 ms for libraries up to 1000 assets.

To evaluate software behavior directly, we benchmarked fresh isolated PixelDeck libraries containing 100, 500, and 1000 image assets imported into clean temporary databases and storage roots. Import duration, throughput, representative query latency, and peak memory usage were recorded (Table 1; and Table 2). Across this series, import throughput exceeded 500 files/min, while representative median query latencies remained below 40 ms for these fresh libraries. Benchmark input characteristics are summarized in Table S1, and overall benchmarking trends are summarized in Fig. 4.

We also revised the related-tools discussion by adding a structured comparison with OMERO, QuPath, Fiji/ImageJ, and BisQue (Table 3). This comparison distinguishes deployment model, server requirements, repository behavior, indexing and search support, duplicate handling, and intended workflow scope. A complementary setup and deployment comparison is provided in Table S2, and Fig. S1 summarizes the qualitative positioning of PixelDeck relative to these representative tools.

To provide a runnable example beyond the main benchmark and comparison analyses, we also assembled a reproducibility package comprising a documented Windows quick-start sequence, a bundled synthetic RGB sample dataset, and verifiable outcomes for a complete local-ingestion workflow. In this package, a 12-image sample set was generated and imported under a unique PixelDeck source label; the initial import completed with 12 discovered and 12 imported assets, source-filtered browse and search each returned 12 assets, and a repeated import produced 12 duplicate events with no new assets. Workflow verification outcomes are summarized in Table S3, and the corresponding timing overview is shown in Fig. S2.

To test whether PixelDeck improves practical local curation workflows rather than merely presenting a convenient architecture, we added workflow case studies at two library sizes. These examples quantified four common user tasks: recovering an imported subset, searching within that subset, re-importing the same folder to verify exact-duplicate handling, and exporting the curated subset as a ZIP archive. Across the 12-image reproducibility library and 100-image companion library, measured PixelDeck task durations ranged from milliseconds to 6.210 s. The corresponding action-count comparison showed fewer required actions for PixelDeck than for the illustrative manual filesystem workflow across all four tasks (Table S4; Table S5; Fig. S3).

### 1.3. Impact

PixelDeck addresses an under-served problem in biomedical imaging workflows: the management of large collections of exported image and video assets once they leave the primary acquisition or analysis environment. Many laboratories rely on conventional filesystem navigation for this stage of work, even when datasets have become too large for convenient manual browsing. This creates bottlenecks in rediscovery, comparison, reuse, and export of previously generated visual outputs. PixelDeck improves this process by providing a lightweight local application that introduces structured metadata, duplicate-aware import, preview generation, full-text search, and interactive curation without requiring a complex server deployment.

The impact of PixelDeck lies primarily in workflow reproducibility and transparent local curation. By normalizing scattered folders into a managed local library, the software makes it easier to recover prior outputs, defined here as rediscovering and reopening previously generated image or video files that were produced before import and then indexed in PixelDeck, rather than re-running the original upstream preprocessing or analysis pipeline. PixelDeck also supports assembling subsets for manuscript preparation, monitoring import provenance, and avoiding unintentional duplication of media assets. The explicit separation of immutable originals from generated browsing derivatives preserves data integrity while improving interface responsiveness. Because metadata are stored in SQLite and media remain visible on disk in a transparent directory layout, the system remains inspectable and easy to back up, migrate, or adapt to new workstation environments.

Although the motivating use cases are histology, fluorescence microscopy, and high-throughput imaging, the software is not restricted to a single biomedical domain. Any research setting that produces large collections of exported images or short videos can use the same import, search, browsing, and export workflow. PixelDeck therefore occupies a useful middle ground between ad hoc filesystem browsing and more infrastructure-heavy image management platforms(6–10). Its main contribution is not domain-specific image analysis, but rather a local software layer for organizing and reusing large visual research collections.

### 1.4. Limitations

The current PixelDeck implementation is designed primarily for local single-user or lightly shared workstation workflows rather than for high-concurrency institutional deployment. Its SQLite-based metadata store is suitable for transparent local management and the benchmarked library sizes reported here, but it is not intended to replace multi-user archive infrastructure built around database servers, centralized authentication, or continuously shared remote storage. Similarly, the current SHA-256 duplicate-detection workflow identifies exact duplicate files and prevents redundant re-import of identical assets, but it does not detect near-duplicates, visually similar images, or derivative variants that differ at the byte level. PixelDeck also does not currently provide multi-user editing, cloud-hosted synchronization, or collaborative server-side access controls. These boundaries reflect the intended scope of the present release: a local-first workstation tool for organizing, browsing, and reusing exported biomedical image and video assets with transparent on-disk storage rather than a cloud-scale or enterprise laboratory information platform.

## 2. Conclusions

PixelDeck is a local-first software system for organizing, browsing, and exporting large image and video libraries on commodity workstations. The software combines a browser-based user interface, REST-style application routes, a SQLite metadata store, managed local media storage, and a dedicated background worker for import and export tasks. This architecture supports duplicate-aware ingestion, metadata extraction, generation of display-oriented derivatives, responsive browsing, and asynchronous export without requiring cloud infrastructure or a complex server environment.

The current implementation is particularly well suited to workflows based on exported histology images, fluorescence microscopy outputs, and high-throughput imaging derivatives stored in common viewable formats. By providing structured indexing, full-text search, preview generation, and curation tools on top of transparent local storage, PixelDeck helps laboratories convert scattered folders into searchable and reusable image libraries. Future development will focus on broader scientific image-format support, richer metadata extraction, improved reproducibility/demo packaging, and more flexible deployment options. Within the limitations discussed above, the present version provides a practical workstation-oriented tool for everyday biomedical image library management.

## Supplementary Material

Supplementary Information

Supplementary material associated with this article can be found, in the online version, at doi:10.1016/j.softx.2026.102803.

## Figures and Tables

**Table 1:** PixelDeck import benchmarks across increasing library sizes.

Library size (images)	Import duration (s)	Import throughput (files/min)	Peak import RSS (MB)
100	8.05	745.73	177.39
500	41.5	722.81	207.43
1000	118.65	505.67	218.99

**Table 2:** Representative PixelDeck query latency benchmarks.

Library size (images)	Task	Median latency (ms)	Mean latency (ms)	Peak RSS (MB)
100	Browse newest	6.484	6.514	147.46
100	Token search	3.355	3.295	149.97
500	Browse newest	32.225	32.481	165.48
500	Token search	7.849	7.832	157.84
1000	Browse newest	35.508	36.69	185.04
1000	Token search	5.999	5.327	190.3

**Table 3:** Structured comparison of PixelDeck with representative related image-management and analysis platforms.

Tool	Deployment model	Server required	Managed repository	Structured search	Duplicate handling	Primary workflow
PixelDeck	Local browser app plus background worker	No	Yes	Yes	Exact duplicates via SHA-256	Local media-library organization for exported biomedical images and videos
OMERO	Client/server platform	Yes	Yes	Yes	Not presented as a core exact-dedup workflow	Server-based scientific image management, metadata, visualization, and annotation
QuPath	Desktop application	No	Project-based, not a general managed media repository	Limited compared with media-library search workflows	No core duplicate-deduplication workflow	Digital pathology viewing, annotation, measurement, and analysis
Fiji/ImageJ	Desktop application	No	No	No repository-level search model	No core media-library deduplication workflow	General image processing and analysis via plugins/macros
BisQue	Server/web platform	Yes	Yes	Yes	Not presented as a core exact-dedup workflow	Web-based bioimage data management and analysis platform
