# Supplementary material for: PixelDeck: A local-first media library manager for biomedical imaging
Source: SoftwareX. Author manuscript; Available in PMC 2026 Jul 9. (PMC13341158; doi:10.1016/j.softx.2026.102803)
Supplement: Supplementary Information [file NIHMS2191697-supplement-Supplementary_Information.pdf]

SUPPLEMENTARY INFORMATION

PixelDeck: A local-first media library manager for biomedical imaging

Benjamin L. Kidder[1-2*]

[1]Department of Oncology, Wayne State University School of Medicine, Detroit, MI, USA

[2]Karmanos Cancer Institute, Wayne State University School of Medicine, Detroit, MI, USA

*Correspondence:
Benjamin L. Kidder
Email : benjamin.kidder@wayne.edu

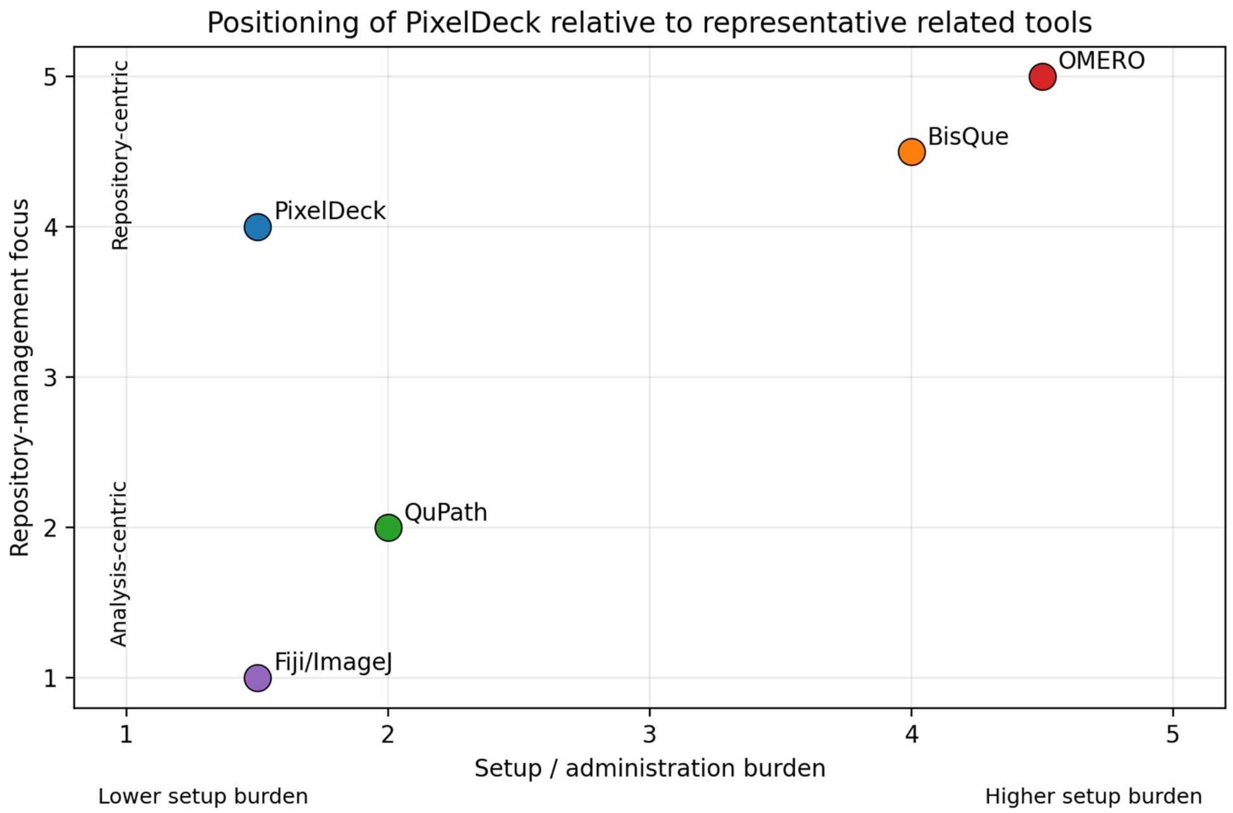

Figure S1. Qualitative positioning of PixelDeck relative to representative related tools. The x-axis summarizes relative setup and administration burden, ranging from lightweight workstation deployment to more demanding server-based installation and maintenance. The y-axis summarizes repository-management focus, ranging from primarily analysis-centric desktop environments to platforms centered on managed image repositories and searchable metadata stores.

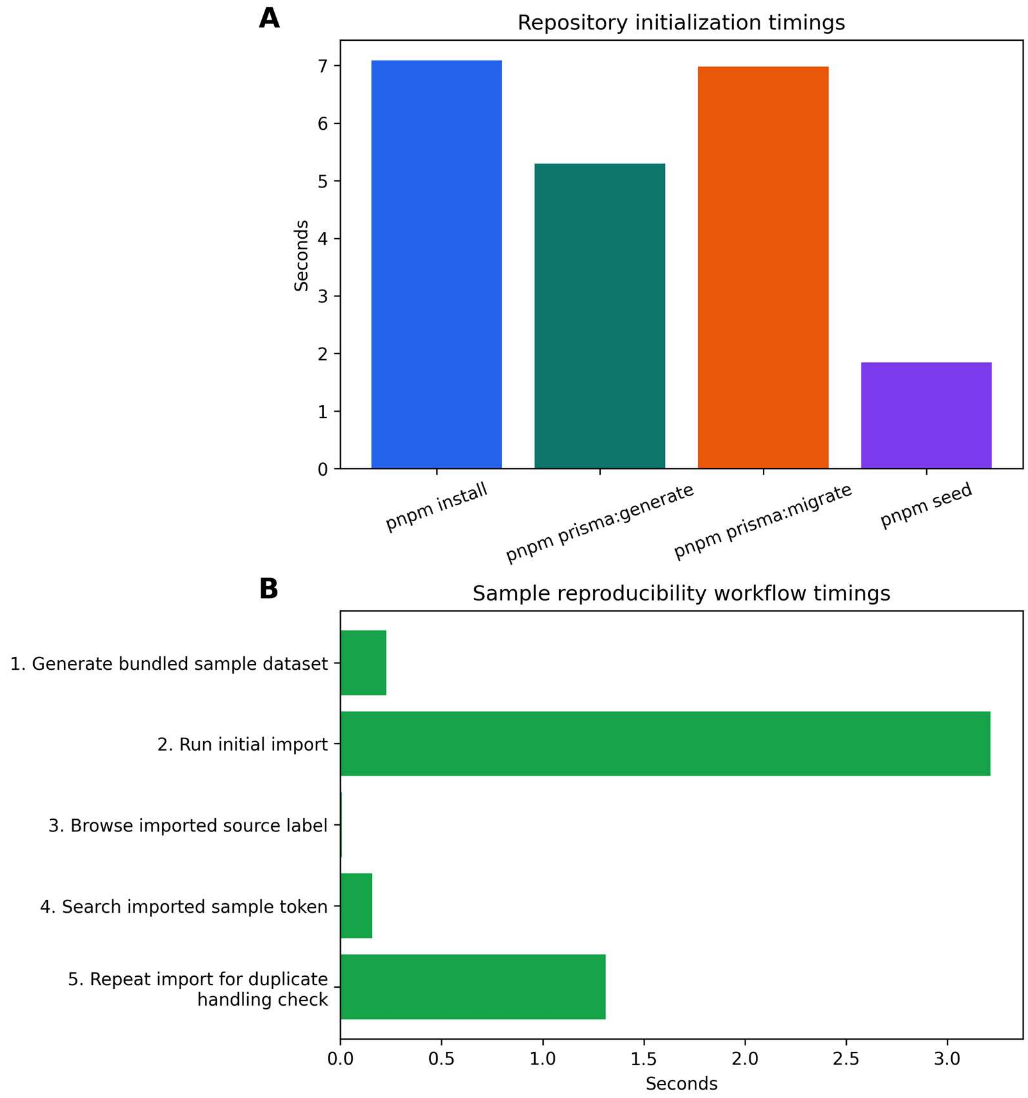

Figure S2. Reproducibility package timings for repository initialization and an example PixelDeck workflow. (A) Observed timings for the documented repository initialization steps (pnpm install, pnpm prisma:generate, pnpm prisma:migrate, pnpm seed) on the current Windows workstation. (B) End-to-end verification workflow timings using a bundled synthetic sample dataset, including dataset generation, initial import, source-filtered browsing, source-filtered search, and duplicate-aware re-import. Table S3 provides the corresponding workflow-verification outcomes.

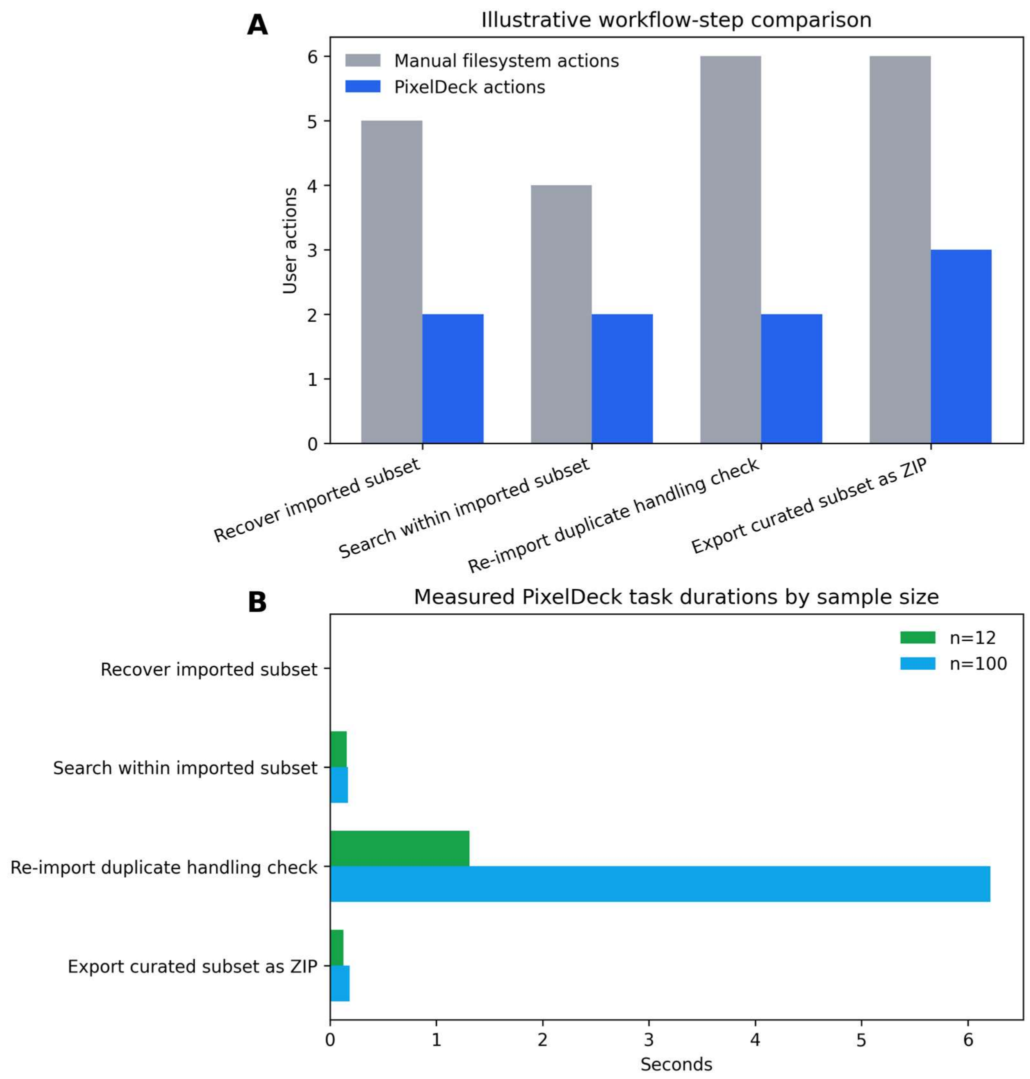

Figure S3. Workflow case studies for local media-curation scenarios at two library sizes. (A) Illustrative action-count comparison between manual filesystem workflows and PixelDeck workflows for four common tasks: recovering an imported subset, searching within the subset, verifying exact-duplicate handling by repeated import, and exporting a curated subset as a ZIP archive. (B) Measured PixelDeck execution times for the same tasks using n = 12 and n = 100 imported assets. Tables S4 and S5 provide the corresponding action-count and timing values.

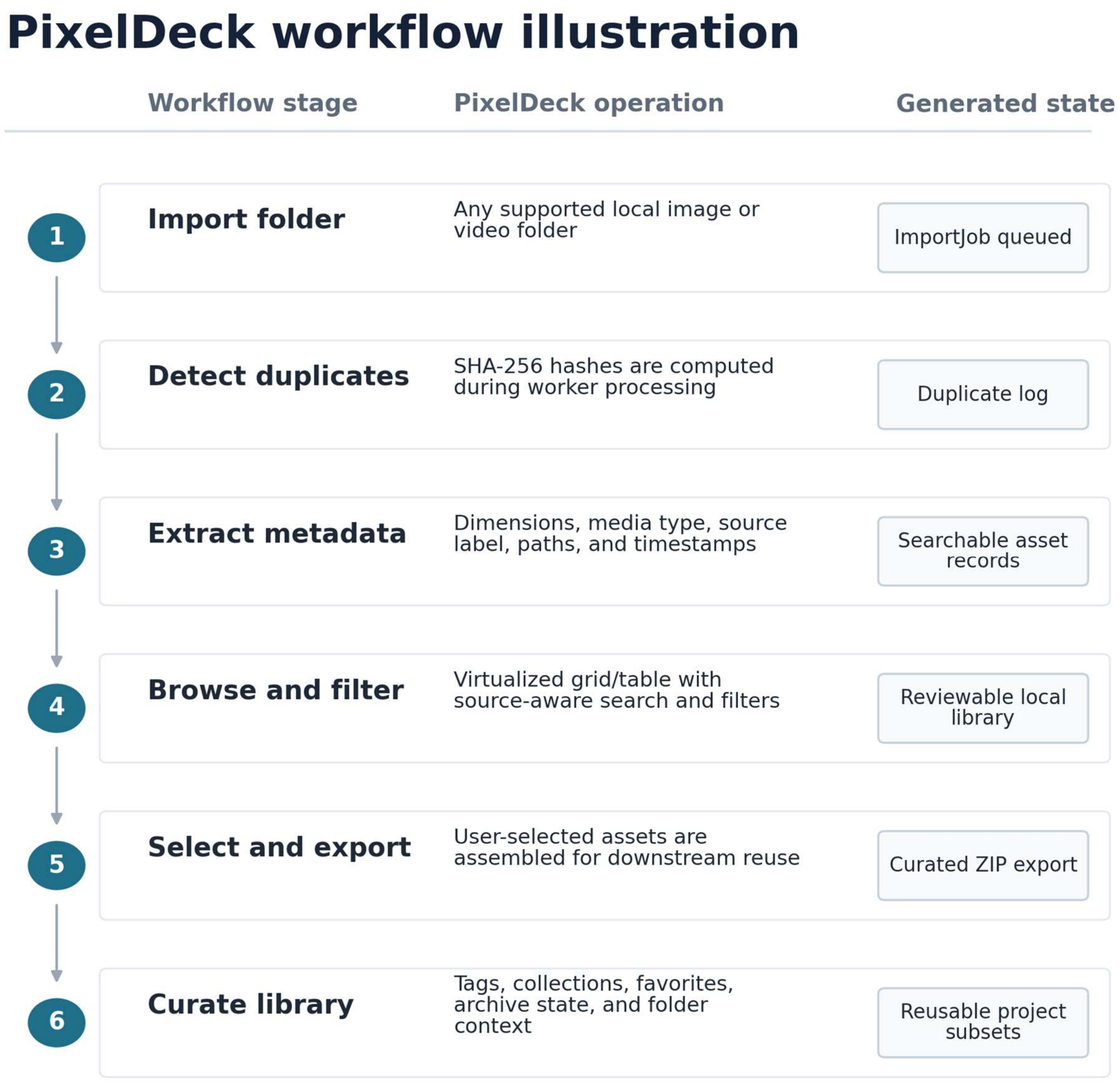

Figure S4. Step-by-step PixelDeck workflow illustration for source-agnostic media ingestion. The diagram summarizes a representative path from importing any supported local image or video folder through exact SHA-256 duplicate detection, metadata extraction, browsing and filtering, selection and ZIP export, and downstream curation actions such as tagging, collection assignment, and folder-context organization. The workflow is independent of dataset source, naming convention, or experimental domain.

Table S1. Benchmark input characteristics for fresh isolated PixelDeck performance runs.

Benchmark size	Mean file size (MB)	Median file size (MB)	Mean width (px)	Mean height (px)	Dominant formats
100	0.66	0.94	1833.74	1377.84	.jpg:96; .png:4
500	0.56	0.45	1513.95	1135.97	.jpg:460; .png:40
1000	0.55	0.42	1338.46	1026.66	.jpg:648; .png:352

Table S2. Setup and deployment comparison for PixelDeck and representative related tools.

Tool	Deployment model	Server required	Setup burden	Admin burden	Managed repository	Background indexing	Structured search	Duplicate handling	Primary workflow
PixelDeck	Local browser app + worker	No	Low	Low	Yes	Yes	Yes	Exact SHA-256 duplicate detection	Local curation and retrieval of exported biomedical media
OMERO	Client/server platform	Yes	High	High	Yes	Yes	Yes	Not a primary exact-deduplication workflow	Institutional image management, metadata, sharing, annotation
QuPath	Desktop application	No	Moderate	Low	Project-based only	Limited	Limited	No primary duplicate-management workflow	Digital pathology viewing, annotation, measurement, analysis
Fiji/ImageJ	Desktop application	No	Low	Low	No	No	No	No primary duplicate-management workflow	General image processing and plugin-based analysis
BisQue	Web/server platform	Yes	High	High	Yes	Yes	Yes	Not a primary exact-deduplication workflow	Bioimage data management and web-based analysis

Note: Setup and administration burden are qualitative categories used to distinguish lightweight workstation installation from server-oriented deployment and maintenance. The comparison is workflow-oriented rather than a direct speed benchmark because the tools target overlapping but non-identical use cases.

Table S3. Example end-to-end reproducibility workflow and observed outcomes for the bundled PixelDeck sample dataset.

Step	Action	Expected outcome	Observed outcome	Duration (s)
1	Generate bundled sample dataset	`12` JPEG sample images created	`12` JPEG images created	`0.227`
2	Run initial import	Import completes with `12` discovered and `12` imported assets	`12` discovered, `12` imported, `0` duplicates, `0` failed	`3.213`
3	Browse imported source label	Library query returns `12` assets for the imported source	`12` assets returned for source `repro-sample-2026-05-10T21-52-39-219Z`	`0.008`
4	Search imported sample token	Search for `sample` returns the imported sample assets	`12` matching assets returned for query `sample` within source `repro-sample-2026-05-10T21-52-39-219Z`	`0.157`
5	Repeat import for duplicate handling check	Second import completes with duplicate count equal to the `12` sample images and no new assets	`12` discovered, `12` duplicates, `0` failed	`1.312`

Table S4. Workflow case study comparing illustrative manual filesystem actions with PixelDeck actions for common local curation tasks.

Task	Illustrative manual filesystem actions	PixelDeck actions	User gain
Recover imported subset	5	2	Source filtering returns the complete imported subset without folder-by-folder navigation.
Search within imported subset	4	2	Indexed token search replaces manual filename inspection across folders.
Re-import duplicate handling check	6	2	Exact duplicates are flagged automatically rather than requiring manual comparison or overwrite decisions.
Export curated subset as ZIP	6	3	Selected assets can be packaged directly as a managed ZIP export instead of being copied and compressed manually.

Table S5. Measured PixelDeck timings and outcomes for local workflow case studies at two library sizes.

Sample size	Task	PixelDeck duration (s)	Measurable outcome
12 images	Recover imported subset	0.008	12 assets returned for the imported source label
12 images	Search within imported subset	0.157	12 assets returned for the query sample
12 images	Re-import duplicate handling check	1.312	12 duplicates detected; library size preserved at 12 assets
12 images	Export curated subset as ZIP	0.127	12 assets exported into a managed ZIP archive
100 images	Recover imported subset	0.006	100 assets returned for the imported source label
100 images	Search within imported subset	0.171	100 assets returned for the query sample
100 images	Re-import duplicate handling check	6.210	100 duplicates detected; library size preserved at 100 assets
100 images	Export curated subset as ZIP	0.185	100 assets exported into a managed ZIP archive
